# Soft Matrix Combined With BMPR Inhibition Regulates Neurogenic Differentiation of Human Umbilical Cord Mesenchymal Stem Cells

**DOI:** 10.3389/fbioe.2020.00791

**Published:** 2020-07-14

**Authors:** Yingying Sun, Ziran Xu, Meijing Wang, Shuang Lv, Haitao Wu, Guangfan Chi, Lisha Li, Yulin Li

**Affiliations:** ^1^The Key Laboratory of Pathobiology, Ministry of Education, College of Basic Medical Sciences, Jilin University, Changchun, China; ^2^Department of Stomatology, First Hospital of Jilin University, Changchun, China

**Keywords:** stiffness, neural differentiation, bone morphogenetic protein receptor, mesenchymal stem cells, hydrogel

## Abstract

Stem cells constantly encounter as well as respond to a variety of signals in their microenvironment. Although the role of biochemical factors has always been emphasized, the significance of biophysical signals has not been studied until recently. Additionally, biophysical elements, like extracellular matrix (ECM) stiffness, can regulate functions of stem cells. In this study, we demonstrated that soft matrix with 1–10 kPa can induce neural differentiation of human umbilical cord mesenchymal stem cells (hUC-MSCs). Importantly, we used a combination of soft matrix and bone morphogenetic protein receptor (BMPR) inhibition to promote neurogenic differentiation of hUC-MSCs. Furthermore, BMPR/SMADs occurs in crosstalk with the integrinβ1 downstream signaling pathway. In addition, BMPR inhibition plays a positive role in maintaining the undifferentiated state of hUC-MSCs on the hydrogel substrate. The results provide further evidence for the molecular mechanisms via which stem cells convert mechanical inputs into fateful decisions.

## Introduction

Various types of stem cells and progenitor cells have the ability to demonstrate the stiffness of extracellular matrix (ECM; Smith et al., 2018). The cytoskeleton, morphology, and migration of these cells all respond to ECM mechanics within a few hours, and then influence cell proliferation, and/or differentiation at following days (Rammensee et al., 2017). It is important that phenotypic changes in cell morphology occur with several hours: myoblast-like elongation on an intermediate stiff gel, osteoblasts expand on a stiff gel, and neuron-like dendritic branching on a soft gel (Lv et al., 2015).

Discher et al. indicated that the culture of mesenchymal stem cells (MSCs) on a very soft surface enhanced neuromorphological changes and the expression of neural genes (Engler et al., 2006). In particular, MSCs implanted in the polyacrylamide gel with a surface modulus of <1 kPa, suggesting the stiffness of brain tissues, highly expression of neural markers, such as neural structural proteins, including neurofilament and β-III tubulin, and microtubule-associated protein 2 (Hadden et al., 2017). Moreover, other studies have demonstrated that the soft surfaces have a neural-inducing effect on some types of stem cells such as induced pluripotent stem cells, adult neural stem cells, and epidermal stem cells (Thompson and Chan, 2016). There are four major types of polymers that are used in mechano-sensitivity studies: polyacrylamide (PAAM), polydimethylsiloxane (PDMS), and polyelectrolyte multilayer films made of synthetic polyelectrolytes, which are mostly employed as 2D culture substrates; whereas the fourth, poly (ethylene glycol; PEG), is used as a 3D hydrogel with cells embedded in it (Her et al., 2013). Some researchers have synthesized type I collagen and hyaluronic acid scaffolds, found that MSCs were likely to differentiate into neuronal lineage in substrate of 1 kPa, while transformed into glial cells in matrix of 10 kPa (Her et al., 2013). Soft matrix has been displayed to increase the neuronal function and neurogenic differentiation (Lv et al., 2015; Mao et al., 2016).

Stem cells are extremely sensitive to their physical environment, but it is not clear how physical stimuli signals are transduced in the cells (Stukel and Willits, 2018). It has been reported that the BMP signaling pathway is a crucial pathway associated with neural induction of stem cells on stiff surfaces, tissue development, and cell differentiation (Thompson and Chan, 2016). Sun et al. substantiated the inhibitory effect of soft poly (dimethylsiloxane) on Smad1/5/8 phosphorylation of human pluripotent stem cells using immunoblots technique. Indeed, it is supported that YAP/TAZ-mediated nuclear accumulation of SMADs regulates stiffness-dependent neural induction of stem cells (Dupont et al., 2011; Sun et al., 2014). Moreover, in these different cellular responses, focal adhesions formed by integrin clustering are considered to modulate several pathways, making them an vital component of substrate-mediated transduction (Stukel and Willits, 2016).

After understanding the process of interaction between human umbilical cord mesenchymal stem cells (hUC-MSCs) and their physical environment, it is possible to control the differentiation direction of hUC-MSCs by the construction of substrates with unique stiffness characteristics. According to our previous work, PAAM prepared by polymerizing cross-linking of acrylamide and bis-acrylamide, are being used to construct substrates of 1–10, 35–38, and 62–68 kPa. In this study, we adopted a method that soft matrix combined with small molecule inhibitor to regulate the neurogenic differentiation of hUC-MSCs, and to investigate the molecular mechanism underlying how biophysical signals are transduced.

## Results

### Matrix Stiffness Regulates the Morphology and Neural Differentiation

**FIGURE 1:**
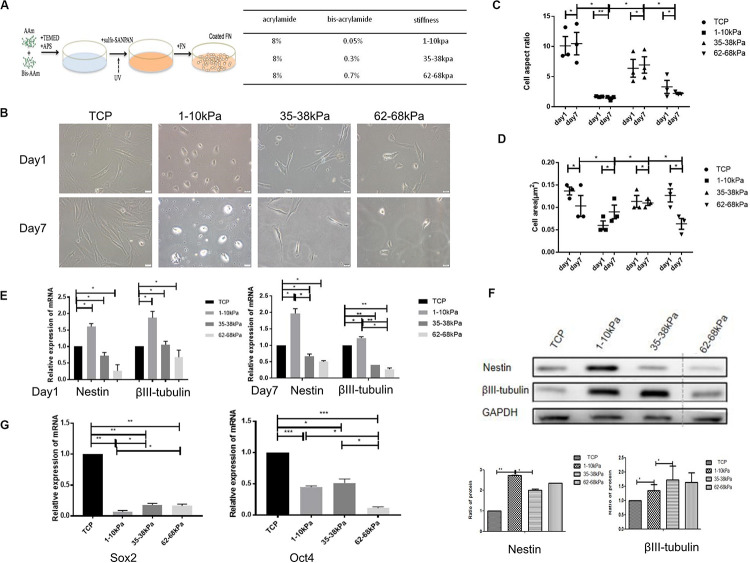
Matrix stiffness regulates cell morphology and neural differentiation ofhuman umbilicalcordmesenchymal stem cells (hUC-MSCs). **(A)** A summary of substrate stiffness construction. Matrixes of different stiffness were prepared by mixing the same concentration of acrylamide (AAm) with different concentrations of bis-acrylamide (Bis-AAm; 0.05, 0.3, and 0.7%). Sulfo-SANPAH (hexanoate) was cross-linked via ultraviolet irradiation and then coated with fibronectin for cell adhesion. **(B)** Matrix stiffness affects the morphological characteristics of hUC-MSCs. Morphological characteristics of hUC-MSCs were observed under a scanning electron microscope at days 1 and 7. TCP group: cells on Tissue Culture Plate. Scale bar = 20 μm. *n* = 3 independent wells. **(C)** The cell aspect ratio was calculated using NIH Image J. The aspect ratio of the cell is the ratio of the major to minor axes (*n* = 3, *p < 0.05,and **p < 0.01). **(D)** The cell area was calculated using NIH Image J (*n* = 3, *p < 0.05,and **p < 0.01). **(E)** qRT-PCR analyses were performed to detect the expression of neuronal-specific markers Nestin and βIII-tubulin in hUC-MSCs at day 1 and day 7 (Results are presented as mean ± SEM, *n* = 5 independent experiments, *p < 0.05, and **p < 0.01). **(F)** Western blotting analysis of Nestin and βIII-tubulin at day 7 (Results are presented as mean ± SEM, *n* = 3, *p < 0.05, and **p < 0.01). **(G)** qRT-PCR analysis of stem cells self-renewal markers SOX2 and OCT4 at day 1 (Results are presented as mean ± SEM, *n* = 5 independent experiments, *p < 0.05, **p < 0.01, and ***p < 0.001).

The morphological characteristics of hUC-MSCs changed dramatically when cultured on matrix gels with different stiffness at days 1 and 7. From the results, comparing day 1 with the control group (cells on Tissue Culture Plate TCP), cells on matrix with 1–10 kPa became two ends shortened and round; cells on 35–38 kPa showed spindle-shaped; while cells on 62–68 kPa were well spread and irregularly shaped, with a few cells still being spindle-shaped. Furthermore, the morphological differences in cells in each group at day 7 were more apparent compared to those at day 1 (Figure 1B).

Subsequently, the aspect ratios (ratio of cell length to width) of hUC-MSCs at day 1 and day 7 were calculated. It is obvious that the aspect ratio in the stiffness groups was significantly lower than that in the control group, at both day 1 and day 7. In the stiffness groups, the length-width ratios of hUC-MSCs were highest on 35–38 kPa, followed by 62–68 kPa, and the lowest on 1–10 kPa. Under the same culture conditions, the cell morphology changed markedly in each stiffness group, which indicated that matrix stiffness affected the morphology of the cells. In addition, there was no significant difference in aspect ratios over time in each group (Figure 1C). Then the cell area of hUC-MSCs was measured at days 1 and 7. The results showed that, at day 1, the area of cells on 1–10 kPa was the smallest, medium on 35–38 kPa, and the largest on 62–68 kPa. Interestingly, the spreading area of these cells was increased with the increasing stiffness. However, at day 7, the spreading area of cells on 62–68 kPa decreased instead. In general, the spreading ability of the cells was higher on 35–38 kPa than that on 1–10 kPa soft matrix and 62–68 kPa stiff matrix. The results showed that the cell area increased gradually on 1–10 kPa over time, but it was not the same on 62–68 kPa, which may be related to cell differentiation (Figure 1D).

Thereafter, the neural stem cell marker (Nestin) and the specific marker in early stage of neurons (βIII-tubulin) were detected using qRT-PCR. It was shown that the expression levels of Nestin and βIII-tubulin were higher in hUC-MSCs on 1–10 kPa than TCP and the other two groups (35–38 kPa and 62–68 kPa). With the stiffness increasing, the expression of neural markers decreased (Figure 1E). It confirms that hUC-MSCs highly expressed neuronal markers on matrix of 1–10 kPa, that is stiffness of 1–10 kPa induced the neural differentiation of hUC-MSCs. Subsequently, the total proteins of hUC-MSCs were collected at day 7, and western blotting was utilized to examine the protein expression levels of Nestin and βIII-tubulin. The results showed that, the cells highly expressed Nestin, and βIII-tubulin on 1–10 kPa compared with the TCP group, which was consistent with the results of the qRT-PCR. It further proved that hUC-MSCs tend to differentiate in neural direction on 1–10 kPa (Figure 1F). However, the expression of βIII-tubulin on 35–38 kPa was higher than that on 1–10 kPa, which conflicted with the result of mRNA detection, which may be affected by many other factors.

Furthermore, mRNA expression of stem cell self-renewal specific genes SOX2 and OCT4 were detected using qRT-PCR. The results showed that SOX2 and OCT4 expression by hUC-MSCs on 1–10, 35–38, and 62–68 kPa were prominently downregulated compared with that of TCP, as well as there was a difference between each group (Figure 1G). These genes included some that are known to participate in the process of self-renewal; those observed indicated that the self-renewal ability of hUC-MSCs decreased, which was induced by stiffness of matrix.

### Soft Matrix Combined With BMPR Inhibition Enhances the Neural Differentiation of hUC-MSCs

**FIGURE 2:**
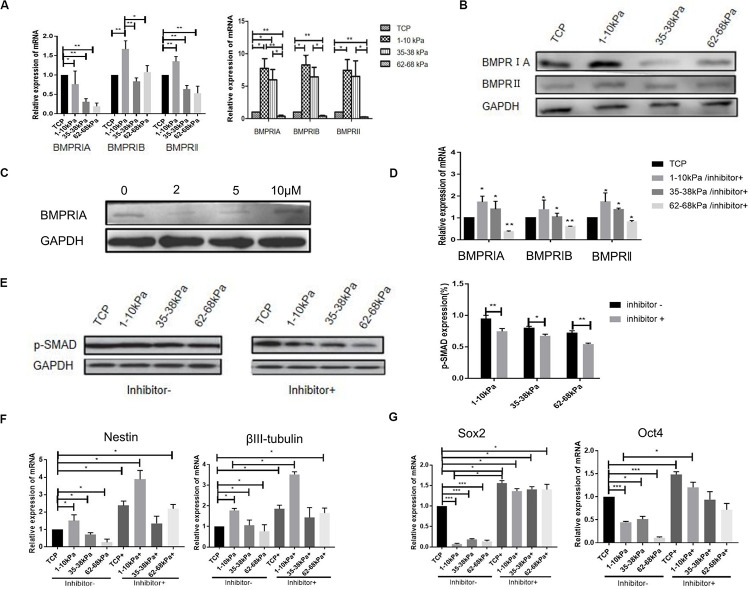
BMPR is involved in stiffness-mediated neural differentiation of hUC-MSCs. **(A)** qRT-PCR detection of BMPR subtypes expression at day 1 and day 7 (Results are presented as mean ± SEM, *n* = 5 independent experiments, *p < 0.05, and **p < 0.01). **(B)** Expression of BMPR subtypes detected using western blotting, and statistical analysis diagram (Results are presented as mean ± SEM, *n* = 3, *p < 0.05, and **p < 0.01). **(C)** The best concentration of BMPR inhibitor was determined using western blotting, the best concentration of inhibitor was 2 μM (*n* = 3 independent experiments). **(D)** The cells were collected at day 7 for detection of BMPR expression after stiffness groups adding inhibitor using RT-qPCR (Results are presented as mean ± SEM, *n* = 5 independent experiments, *p < 0.05, and **p < 0.01). **(E)** Expression of p-SMAD was detected using western blotting before and after BMPR inhibition, and statistical analysis diagram (Results are presented as mean ± SEM, *n* = 3, *p < 0.05, and **p < 0.01). **(F)** qRT-PCR analysis of Nestin and βIII-tubulin expression after BMPR inhibition for 24 h. Statistical analysis was performed using one-way ANOVA. Statistical significance was defined as *p* < 0.05. Results are presented as mean ± SEM (*n* = 5 independent experiments, *p* < 0.05). **(G)** qRT-PCR analysis of SOX2 and OCT4 expression after BMPR inhibition for 24 h. Statistical analysis was performed using one-way ANOVA. Statistical significance was defined as *p* < 0.05. Results are presented as mean ± SEM (*n* = 5 independent experiments, *p* < 0.05, and ***p < 0.001).

The mRNA expression of bone morphogenetic protein receptor (BMPR; BMPRIA, BMPRIB, and BMPRII) was detected using qRT-PCR after cells were cultured for 24 h and 7 days. The highest expression level of BMPR was found on matrix of 1–10 kPa. BMPRIA was 7.9-fold higher, BMPRIB was 8.5-fold higher, and BMPRII was 7.5-fold higher than that of the control group. In addition, the expression of BMPR subtype decreased gradually with increasing stiffness, and there was a difference between each group (Figure 2A). Moreover, BMPR (BMPRIA, BMPRIB, and BMPRII) expression in the cells at the protein level was detected using western blotting at day 7. Results showed that, the expression of BMPRIA in hUC-MSCs was significantly higher than that of the TCP group, with a statistically significant difference. However, the expression of BMPRII on 1–10 kPa was the lowest (Figure 2B). It has been reported that BMPRIA is involved in the neural differentiation which is regulated by stiffness, therefore we investigated the role of BMPRIA in the following experiments (Du et al., 2011; Xu et al., 2014).

To further explore the role of BMPR, we selected BMPR inhibitor (LDN-193189) to inhibit BMPR. The concentration gradient of BMPR inhibitor was 0, 2, 3, 5, 10, and 15 μM according to previous reports (Yu et al., 2008; Al Madhoun et al., 2016). The best concentration of BMPR inhibitor was detected using western blotting. Under a microscope, the cell death rate was higher when the concentration of the inhibitor was 10 and 15 μM, therefore, the concentration of the inhibitor was 0, 2, 3, and 5 μM in this study. When the concentration of the inhibitor was 2 μM, BMPR expression level in hUC-MSCs was significantly inhibited compared with that of the non-inhibitor group (Figure 2C), thus the concentration of the inhibitor was 2 μM in subsequent experiments. Next, BMPR expression in cells, after inhibition for 7 days, was detected using qRT-PCR. It was shown that after adding inhibitor, the expression of BMPR in hUC-MSCs on 1–10 kPa was still higher than that on TCP (the control group without inhibitor), but it was significantly lower than that without inhibitor at day 7, which proved that BMPR was effectively suppressed (Figure 2D). Furthermore, the expression of BMPR downstream protein p-SMAD was detected using western blotting after adding BMPR inhibitor for 7 days. We found that the expression of p-SMAD was downregulated in all groups after BMPR inhibition and decreased with increasing stiffness (Figure 2E).

Next, the expression levels of Nestin and βIII-tubulin in hUC-MSCs after BMPR inhibition were detected. It is found that the expression of Nestin and βIII-tubulin were both significantly upregulated after BMPR inhibition in all groups (Figure 2F). Moreover, the expression of neural markers in 1–10 kPa was the highest in different stiffness groups after BMPR inhibition (Figure 2F). It proved that soft matrix of 1–10 kPa combined with BMPR inhibition can further enhance the ability of hUC-MSCs neural differentiation. In addition, we detected the mRNA expression of stem cell self-renewal specific genes SOX2 and OCT4 after BMPR inhibition using qRT-PCR. It was shown that SOX2 and OCT4 expression in hUC-MSCs in all groups was prominently upregulated compared to those not inhibited, and there was a difference between each group (Figure 2G). It indicated that the self-renewal ability of hUC-MSCs on the hydrogel matrix was increased after BMPR inhibition. From these results, we conclude that the 1–10 kPa soft matrix promotes the neurogenic differentiation of hUC-MSCs, and BMPR may play a negative role during this process. In addition, compared with the TCP group, the hydrogel stiffness regulates the directional differentiation of hUC-MSCs, and when BMPR is inhibited, the characteristics of hUC-MSCs are maintained and the differentiation is inhibited, indicating that BMPR has a positive regulatory effect on the characteristics of hUC-MSCs in each stiffness group.

### BMPR Has Crosstalk With Integrin Pathway in Soft Matrix Inducing Neural Differentiation of hUC-MSCs

**FIGURE 3:**
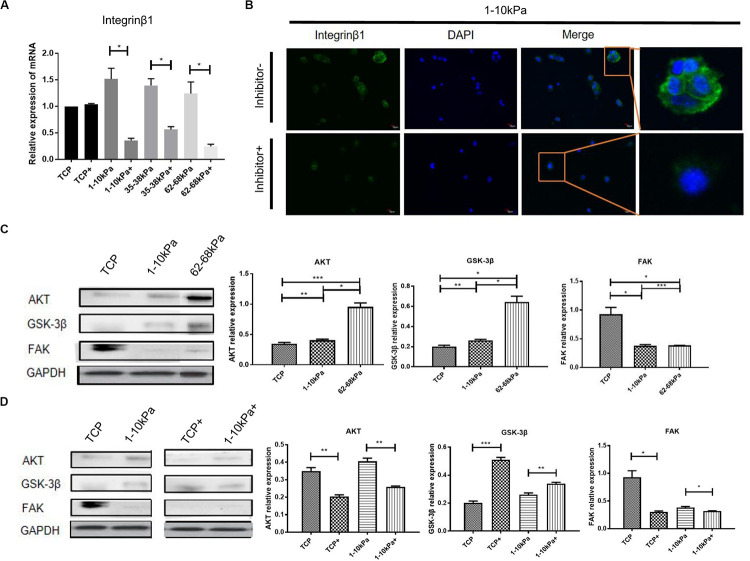
The downstream molecules of integrinβ1 were detected after BMPR inhibition. **(A)** qRT-PCR analysis of integrinβ1 after BMPR inhibition for 24 h. Results are presented as mean ± SEM (*n* = 5 independent experiments, *p < 0.05, and **p < 0.01). “+”represents the group contained inhibitor. **(B)** Staining for integrinβ1 in hUC-MSCs, which were treated on 1–10 kPa before and after BMPR inhibition for 24 h, and then observed under a confocal microscope (Olympus FV 1200; *n* = 3 independent experiments, scale bars, 10 μm). **(C)** Western blotting detection and statistical analysis diagram showing proteins associated with integrinβ1 including AKT, GSK-3β, and FAK on TCP, 1–10 kPa, and 62–68 kPa before adding inhibitor (Results are presented as mean ± SEM, *n* = 3, *p < 0.05, **p < 0.01, and ***p < 0.001). **(D)** Western blotting detection of expression of AKT, GSK-3β, and FAK on TCP and 1–10 kPa after adding inhibitor (Results are presented as mean ± SEM, *n* = 3, *p < 0.05, **p < 0.01, and ***p < 0.001). “+” represents the group contained inhibitor.

To detect the changes of integrin pathway in the process that soft matrix promotes the neural differentiation of hUC-MSCs, we detected the mRNA expression of integrinβ1 after BMPR inhibition for 24 h using qRT-PCR. It was shown that, integrinβ1 expression in hUC-MSCs after BMPR inhibition in three stiffness groups decreased prominently compared with those not inhibited, and there was a difference between each group (Figure 3A). Then, we focused on 1–10 kPa and performed immunofluorescence staining of integrinβ1 at 24 h. The results showed that integrinβ1 was mainly distributed on the cell membrane before BMPR inhibition. Moreover, integrinβ1 expression decreased significantly after BMPR inhibition (Figure 3B). In addition, total hUC-MSCs proteins were collected at day 7, and the levels of integrin related proteins FAK, AKT, and GSK-3β were detected using western blotting. Comparing the three groups of TCP, 1–10 kPa soft matrix and 62–68 kPa stiff matrix, the protein expression levels of AKT and GSK-3β in the two stiffness groups were higher than those in the TCP, representing the original state of cells, and increased with increasing stiffness; while the level of FAK in the stiffness groups was lower than that in the TCP, but there was no significant difference between the stiffness groups (Figure 3C). This indicates that at this point of time, AKT and GSK-3β were involved in stiffness regulating the directional differentiation of hUC-MSCs, whereas FAK did not play an important role at this time. Next, we compared the levels of integrin related signaling proteins before and after BMPR inhibition in the TCP and 1–10 kPa groups. The results showed that in the TCP group, the levels of AKT and FAK decreased after BMPR inhibition, while the level of GSK-3β increased (Figure 3D). It indicates that BMPR inhibitor decreased the levels of AKT and FAK but suppressed the level of GSK-3β in the primitive state of hUC-MSCs. In the 1–10 kPa group, after adding BMPR inhibitor, the level of AKT was downregulated and GSK-3β was upregulated, while the level of FAK showed no significant change (Figure 3D). This suggests that AKT and GSK-3β cooperated with BMPR to be involved in the soft matrix promoting neural differentiation of hUC-MSCs, while FAK was not involved in this regulatory process at this point.

## Discussion

In recent years, increasing attention has been given to the application of hUC-MSCs in cell therapy (Shang et al., 2017). Three properties make MSCs optimal for tissue regeneration:immunoregulatory capacity beneficial to alleviate abnormal immune responses; paracrine or autocrine functions that generate growth factors, and the ability to differentiate into target cells (Han et al., 2019). This study adopted a new method to promote neural differentiation of hUC-MSCs *in vitro* and laid a solid theoretical foundation for the design of regenerated materials in tissue engineering. In this study, polyacrylamide hydrogel was successfully constructed to imitate the stiffness of brain and bone tissues, through gradient elasticity of 1–10, 35–38, and 62–68 kPa. Although soft matrix is able to induce stem cells to neural differentiation, the stiffness is not the same for different matrix materials (Tse and Engler, 2011; Oh et al., 2016). The stiffness of 1–10 kPa was selected in this experiment according to our previous study.

These results suggest that soft matrix regulates neural differentiation of hUC-MSCs,and small molecule inhibition of BMPR can improve neural differentiation of stem cells. Small-molecule LDN-193189, a selective inhibitor of BMP type I receptors, can selectively suppress the activities of receptor-like kinases (ALK2, ALK3, and ALK6; Han et al., 2015; Chiba et al., 2017). Additionally, BMP ligands can promote the phosphorylation and activation of BMP type I receptors by BMP type II receptors (BMPRII, ActRIIA, and ActRIIB; Miyazono et al., 2010). The activated BMP type I receptors phosphorylate BMP signaling effectors SMAD1/5/8, which regulates gene transcriptions through the transport to the nucleus (Yu et al., 2008). Studies have shown that SMADs localization can be transferred from the nucleus to the cytoplasm when MSCs differentiation is induced on stiff or soft substrates (Du et al., 2011). Therefore, from this study we concluded that BMPR inhibition prevented the entry of SMADs into the nucleus, which further promoted neural differentiation of hUC-MSCs.

Importantly, our results have also proved the synergistic effect of BMPR and integrinβ1 on soft matrix inducing neural differentiation of hUC-MSCs. It has been demonstrated that integrinβ1 is the key molecule in the process of cell sensing matrix stiffness (Jiang et al., 2016; Desai et al., 2019). Soft ECM can promote the neural differentiation of BM-MSCs through the endocytosis of integrinβ1 (Du et al., 2011). In addition, the co-localization of BMPRIA and integrinβ1 results in receptor internalization, which in turn regulates downstream SMADs phosphorylation and nuclear translocation, thus regulating cell fate (Provenzano et al., 2009; Shih et al., 2011). Based on these data, the observations in this study are consistent with previous reports. In summary, we demonstrated that not only is BMPR involved in the process of MSC differentiation induced by soft matrix, but also there is crosstalk between BMP signaling pathway and integrin-related AKT, GSK-3β signaling. It is reported that MSCs respond to mechanical stimuli through activation of FAK and AKT pathways by vibration induced RhoA signaling, F-actin remodeling, and repression of adipogenic gene expression (Graham and Burridge, 2016). However, further study is needed on how these protein molecules interact with each other.

Understanding how soft surfaces affect molecular signaling pathways could help us to design the materials that strengthen neural differentiation; thus better design surfaces that promote neural function. However, the cells are in a multifactorial internal environment *in vivo*. Therefore, further researches are needed to be conducted, such as neural differentiation was induced by the combination of soft surfaces and soluble factors, to mimic the ECM environment of cells *in vivo*, thus promoting later differentiation into specific subtypes of neurons.

## Materials and Methods

### Cell Culture and Identification

The culture and identification of hUC-MSCs were based on our previous research methods (Xu et al., 2017). hUC-MSCs were used for all experiments. All experimental procedures were approved by the ethics committee of Jilin University and conformed to the regulatory standards.

### Fabrication of Polyacrylamide Matrix With Gradient Stiffness

Matrixes with gradient stiffness were prepared as previous reports (Engler et al., 2006; Sun et al., 2018). The concentrations of acrylamide (AAm; Sigma-Aldrich, St. Louis, MO, United States) and bis-acrylamide (Bis-AAm; 0.05, 0.3, and 0.7%; Sigma-Aldrich) are shown in Figure 1A.

### Cell Culture on the Hydrogel and Analysis of Cell Morphology

Human umbilical cord mesenchymal stem cells were passaged 4–6, and then seeded on 6-well plates (1–1.5 × 10^5^/cm^2^ cells) with hydrogel matrix. The growth medium was changed every 3 days. NIH ImageJ was utilized to compute the major and minor axes of the cells based on the binary image of the cells. The aspect ratio of the cell was the ratio of the major to minor axes.

### Gene Expression Assay

Total RNA was isolated with TRI reagent (Takara, Tokyo, Japan), and then a PrimeScript RT reagent kit (Takara) was used to synthesize first strand cDNA. Afterwards, qRT-PCR was applied to examine the relative expression of related genes. The sequences of all primers were shown in Table 1.

**TABLE 1:** Primer sequences for qRT-PCR.

**Gene**	**Forward (5′ to 3′)**	**Reverse (5′ to 3′)**
BMPRIA	TCTCAAGCAGACGTCGTTAC	CCGGACCATCTGAATCTGTT
BMPRIB	ACCACTCAGTCCACCTCATT	CGGTCTCCTGTCAACATTCT
BMPRII	TGCAGATGGACGCATGGAA	AGCTTACCCAGTCACTTGTGTGGAG
βIII-tubulin	GGAGATCGTGCACATCCAG	TCGATGCCATGCTCATCAC
Nestin	GCGTTGGAACAGAGGTTGGA	TGGGAGCAAAGATCCAAGAC
Integrinβ1	TGCCAGCCAAGTGACATAGAGA	ATCCGTTCCAAGACTTTTCACAT
SOX2	ACATGAACGGCTGGAGCA A	GTAGGACATGCTGTAGGTGGG
OCT4	CTGGGTTGATCCTCGGACCT	CACAGAACTCATACGGCGGG
Actin	TGCCATCCTAAAAGCCAC	TCAACTGGTCTCAAGTCAGTG

### Detection of Integrinβ1 Using Immunofluorescence Staining

Human umbilical cord mesenchymal stem cells were counted and seeded on a 24-well culture plate with different stiffness at day 1, rinsed with PBS 3 times. The cells were then fixed with 4% paraformaldehyde for 15 min, and blocked with 1% bovine serum albumin (BSA) for 1 h. Anti-integrinβ1 was prepared with 0.5% BSA, 300 μl was added to each well, and then stored at 4°C overnight. After incubation of the secondary antibodies for 1 h, Hoechst33342 (proportion 1:10000) was prepared and used to dye the nucleus for 10–15 min. After rinsing with PBS, the cells were observed under a laser scanning confocal microscope.

### Western Blotting

The proteins were extracted from cells using buffer containing protease and phosphatase inhibitors (Dingguo, Beijing, China). The proteins were separated by electrophoresis on 8% polyacrylamide gels, and then transferred to polyvinylidene fluoride (PVDF) membranes. Afterwards, the membranes were blocked and probed with antibodies for BMPRIA (Abcam), BMPRIB (Abcam), BMPRII (Abcam), GAPDH (CST), Nestin (CST), βII-tubulin (CST), GSK-3β (CST), and FAK (CST) overnight at 4°C. The secondary antibodies were incubated with the membranes on the next day. Finally, the protein bands were visualized using chemiluminescence.

### Statistical Analysis

Descriptive analysis was performed to describe the characteristics of the variables. Statistical analysis was performed with the Student’s unpaired *t*-test and one-way ANOVA. Statistical significance was defined as *p* < 0.05. At least three independent experiments were performed in all cases.

## Data Availability Statement

The raw data supporting the conclusions of this article will be made available by the authors, without undue reservation, to any qualified researcher.

## Ethics Statement

The studies involving human participants were reviewed and approved by the First Hospital of Jilin University Ethics Committee (No. 2019–177). The patients/participants provided their written informed consent to participate in this study.

## Author Contributions

YS, LL, and YL were primarily involved in drafting the article. YS performed the experiments and prepared the figures. LL and YL revised the draft manuscript and provided insights. All authors contributed to the writing of the manuscript, and read and approved the final version.

## Conflict of Interest

The authors declare that the research was conducted in the absence of any commercial or financial relationships that could be construed as a potential conflict of interest.
